# Seroprevalence of SARS-CoV-2 antibodies in people with an acute loss in their sense of smell and/or taste in a community-based population in London, UK: An observational cohort study

**DOI:** 10.1371/journal.pmed.1003358

**Published:** 2020-10-01

**Authors:** Janine Makaronidis, Jessica Mok, Nyaladzi Balogun, Cormac G. Magee, Rumana Z. Omar, Alisia Carnemolla, Rachel L. Batterham

**Affiliations:** 1 UCL Centre for Obesity Research, Division of Medicine, University College London, London, United Kingdom; 2 Bariatric Centre for Weight Management and Metabolic Surgery, University College London Hospital, London, United Kingdom; 3 National Institute of Health Research, UCLH Biomedical Research Centre, London, United Kingdom; 4 Department of Statistical Science, University College London, London, United Kingdom; Guy's and Saint Thomas' NHS Foundation Trust, UNITED KINGDOM

## Abstract

**Background:**

Loss of smell and taste are commonly reported symptoms associated with coronavirus disease 2019 (COVID-19); however, the seroprevalence of severe acute respiratory syndrome coronavirus 2 (SARS-CoV-2) antibodies in people with acute loss of smell and/or taste is unknown. The study aimed to determine the seroprevalence of SARS-CoV-2 antibodies in a community-based population with acute loss of smell and/or taste and to compare the frequency of COVID-19 associated symptoms in participants with and without SARS-CoV-2 antibodies. It also evaluated whether smell or taste loss are indicative of COVID-19 infection.

**Methods and findings:**

Text messages, sent via primary care centers in London, United Kingdom, invited people with loss of smell and/or taste in the preceding month, to participate. Recruitment took place between 23 April 2020 and 14 May 2020. A total of 590 participants enrolled via a web-based platform and responded to questions about loss of smell and taste and other COVID-19–related symptoms. Mean age was 39.4 years (SD ± 12.0) and 69.1% (*n* = 392) of participants were female. A total of 567 (96.1%) had a telemedicine consultation during which their COVID-19–related symptoms were verified and a lateral flow immunoassay test that detected SARS-CoV-2 immunoglobulin G (IgG) and immunoglobulin M (IgM) antibodies was undertaken under medical supervision. A total of 77.6% of 567 participants with acute smell and/or taste loss had SARS-CoV-2 antibodies; of these, 39.8% (*n* = 175) had neither cough nor fever. New loss of smell was more prevalent in participants with SARS-CoV-2 antibodies, compared with those without antibodies (93.4% versus 78.7%, *p* < 0.001), whereas taste loss was equally prevalent (90.2% versus 89.0%, *p* = 0.738). Seropositivity for SARS-CoV-2 was 3 times more likely in participants with smell loss (OR 2.86; 95% CI 1.27–6.36; *p* < 0.001) compared with those with taste loss. The limitations of this study are the lack of a general population control group, the self-reported nature of the smell and taste changes, and the fact our methodology does not take into account the possibility that a population subset may not seroconvert to develop SARS-CoV-2 antibodies post-COVID-19.

**Conclusions:**

Our findings suggest that recent loss of smell is a highly specific COVID-19 symptom and should be considered more generally in guiding case isolation, testing, and treatment of COVID-19.

**Trials registration:**

ClinicalTrials.gov NCT04377815

## Abstract 2

In a UK-based cohort, Janine Makaronidis and colleagues investigate COVID-19 seropositivity in individuals with loss of smell and/or taste.

## Author summary

**Why was this study done?:**

- Coronavirus disease 2019 (COVID-19), an infectious disease caused by severe acute respiratory syndrome coronavirus 2 (SARS-CoV-2) virus, was declared a pandemic in March 2020.
- COVID-19 can cause loss or reduced ability to smell (anosmia) or taste, without cough or fever, but few countries recommend self-isolation and testing on the basis of smell or taste changes alone.
- This study aimed to find out the proportion of people who have developed SARS-CoV-2 antibodies in a community-based population with a newly developed loss in their sense of smell and/or taste in London, UK.

**What did the researchers do and find?:**

- Text messages were sent out to people registered with a number of primary care centers in London inviting people with a new loss in their sense of smell and/or taste to participate.
- Recruited participants completed online questionnaires regarding demographics, their loss of smell and/or taste, and other COVID-19 symptoms, before they had a telemedicine consultation with a healthcare professional who confirmed the history of their symptoms and supervised a test to find out if they had SARS-CoV-2 antibodies.
- A total of 78% of 567 people with smell and/or taste loss had SARS-CoV-2 antibodies; of these, 40% had neither cough nor fever, and participants with loss of smell were 3 times more to have SARS-CoV-2 antibodies, compared with those with loss of taste.

**What do these findings mean?:**

- Loss of smell is a highly specific symptom of COVID-19.
- COVID-19 can present with loss of smell and/or taste without cough or fever.
- Loss of smell should be take into consideration in case isolation, testing, and treatment strategies for COVID-19.

## Introduction

Coronavirus disease 2019 (COVID-19) is an acute infectious disease caused by the severe acute respiratory syndrome coronavirus 2 (SARS-CoV-2). COVID-19 has spread exponentially, with 27,417,497 cases and 894,241 deaths reported from 216 countries by 9 September 2020 [1]. In the absence of a vaccine and disease-specific treatments, strategies to contain the pandemic are focused on rapid case isolation and testing. Although originally described as a primarily respiratory disease, reports of COVID-19 presenting with other multisystem symptoms, including loss of smell and taste, emerged rapidly. Understanding the symptomatology of COVID-19 and the predictive value of symptoms is crucial for containment strategies. As lockdown policies ease globally, early recognition of COVID-19 symptoms by the public together with rapid self-isolation and testing will be of vital importance to limit disease spread.

Reports linking loss of the sense of smell and/or taste to COVID-19 emerged in March 2020 [2–4]. SARS-CoV-2 enters the human body via the angiotensin-converting enzyme-2 (ACE-2) receptor, highly expressed in the nasal epithelium [5]. Consequent inflammatory changes in the olfactory neuroepithelium could disrupt olfactory neuron function, leading to smell loss [6,7]. Thus, from a pathophysiological perspective, it is logical for COVID-19 to impact smell [5,6]. Smell and taste are highly interlinked, with an element of taste (flavor) perception mediated through retronasal olfaction; hence, loss of smell results in taste changes [6]. However, within the oral cavity, ACE-2 is highly expressed on tongue epithelial mucosal cells [5], raising the possibility that taste loss results from a direct effect of SARS-CoV-2 on the tongue and that taste loss alone could occur in the absence of smell loss.

Available data suggest a prevalence of smell and/or taste loss in the range of 31%–85% in COVID-19 patients [8–10]. In a prospective epidemiological study, 85.6% and 88.0% of patients with a polymerase chain reaction (PCR)-confirmed COVID-19 diagnosis reported a loss of their sense of smell and taste, respectively [9].

The largest data set of potential COVID-19 related symptoms stems from a web-based app that collected self-reported symptoms from 2,618,862 users in the United Kingdom (UK) and the United States (US). This included the question “Do you have a loss of smell/taste?”. The authors reported a strong association between loss of smell/taste and a diagnosis of COVID-19, and as a consequence, loss of smell/taste are now recognized presentations of COVID-19 in the UK [11,12]. Their methodology did not, however, enable them to differentiate between smell and taste loss [12,13].

The importance of acute loss of smell or taste, in isolation or combination, as a predictor of COVID-19 in a population presenting with chemosensory symptoms is unknown. Currently, recommendations for self-isolation and testing based upon acute loss of smell/taste have only been adopted by a limited number of countries; the majority are focused on fever and respiratory symptoms (Table 1). We therefore set out to quantify the seroprevalence of SARS-CoV-2 specific antibodies in a community-based cohort with a new loss of their sense of smell and/or taste during the COVID-19 outbreak in London, UK. Additionally, we compared the effect of isolated loss of smell and isolated loss of taste separately and then in combination and investigated whether new smell and/or taste loss are indicative of COVID-19 in our study population.

**Table 1:** Recognition of smell and/or taste loss as symptoms of COVID-19 in the 30 countries with the highest number of reported cases globally.

Country	Cases^1^	Deaths^1^	Recognition of smell/taste loss as COVID-19 symptoms	Reference^2^
United States of America	6,328,099	186,699	Yes	https://www.cdc.gov/coronavirus/2019-ncov/symptoms-testing/symptoms.html
India	4,370,128	73,890	No	https://www.mygov.in/covid-19
Brazil	4,162,073	127,464	Yes	https://coronavirus.saude.gov.br/
Russia	1,037,526	18,080	No	https://covid19.rosminzdrav.ru/
Peru	691,575	29,976	No	https://www.gob.pe/8665-sintomas-del-coronavirus-conocer-si-puedo-haber-contraido-el-covid-19
Colombia	679,181	21,813	No	https://d2jsqrio60m94k.cloudfront.net/
Mexico	642,860	64,484	No	https://coronavirus.gob.mx/
South Africa	640,441	15,086	No	https://sacoronavirus.co.za/
Spain	534,513	29,594	Yes	https://www.mscbs.gob.es/profesionales/saludPublica/ccayes/alertasActual/nCov-China/img/COVID19_sintomas.jpg
Argentina	500,034	10,405	Yes	https://www.argentina.gob.ar/coronavirus/glosario/caso-sospechoso
Chile	425,034	11,682	No	https://www.minsal.cl/nuevo-coronavirus-2019-ncov/
Iran	393,425	22,669	No	https://test.corona.ir/coronaTest
France	373,718	30,770	Yes	https://www.gouvernement.fr/info-coronavirus/comprendre-la-covid-19
United Kingdom	354,934	41,675	Yes	https://www.nhs.uk/conditions/coronavirus-covid-19/check-if-you-have-coronavirus-symptoms/
Bangladesh	331,078	4,593	No	https://corona.gov.bd/faq
Saudi Arabia	322,237	4,137	No	https://www.moh.gov.sa/en/HealthAwareness/EducationalContent/PublicHealth/Pages/corona.aspx
Pakistan	299,659	6,359	Yes	http://covid.gov.pk/
Turkey	283,270	6,782	No	https://covid19.saglik.gov.tr/TR-66300/covid-19-nedir-.html
Italy	280,153	35,563	Yes	http://www.salute.gov.it/portale/nuovocoronavirus/dettaglioFaqNuovoCoronavirus.jsp?lingua=english&id=230#1
Iraq	269,578	7,657	No	https://moh.gov.iq/
Germany	255,626	9,342	Yes	https://www.zusammengegencorona.de/en/inform/recognize-symptoms/#faqitem=5750383b-e61b-5792-afac-f36cf48b2a7f
Philippines	245,143	3,986	Yes	https://www.covid19.gov.ph/
Indonesia	203,342	8,336	No	https://www.kemkes.go.id/index.php?lg=LN02
Ukraine	146,511	3,034	No	https://covid19.gov.ua/en
Israel	138,719	1,040	Yes	https://govextra.gov.il/ministry-of-health/corona/corona-virus-en/corona-symptoms-en/
Canada	135,757	9,203	Yes	https://www.canada.ca/en/public-health/services/diseases/2019-novel-coronavirus-infection/symptoms.html
Bolivia	122,308	7,097	No	https://www.boliviasegura.gob.bo/covid-19.php
Qatar	120,579	205	No	https://www.moph.gov.qa/english/Pages/Coronavirus2019FAQs.aspx
Ecuador	110,757	10,627	No	https://www.salud.gob.ec/coronavirus-covid-19/
Kazakhstan	106,498	1,634	No	https://www.gov.kz/memleket/entities/dsm/activities/6626?lang=en&parentId=6625

^1^ Reported cases as per John Hopkins University of Medicine Coronavirus Resource Centre [14], accessed 9 September 2020.

^2^ Accessed 9 September 2020.

COVID-19, coronavirus disease 2019.

## Methods

### Study design

The study was conducted in London, UK, and recruited between 23 April 2020 and 14 May 2020 at a time when loss of smell and/or taste were not recognized as COVID-19 symptoms. Recruitment was timed to capture people who experienced symptoms during the peak of the COVID-19 outbreak in London. Antibody testing was delivered using a telemedicine consultation. This approach was chosen to capture positive cases without the limitations of the time window restrictions of PCR and to enable testing and participation without face-to-face contact, reducing the infection risk to both participants and researchers.

Adults registered with 4 participating primary care centers in London were sent text messages to their mobile telephones inviting those who experienced a new loss of their sense of smell and/or taste in the preceding month to participate. The text message read: “Has your sense of smell or taste reduced in the last month? If yes and you’d like to be part of a COVID-19 research study go to [link]”. Participants were then directed to an online platform (hosted by Dendrite Clinical Systems) with the study information and an eligibility check. Inclusion criteria were age ≥18 years, proficiency in written and spoken English, and access to video-calling. Exclusion criteria were any preexisting loss of the sense of smell or taste of longer than a month’s duration. Participation was voluntary, and written informed consent was obtained electronically. Enrolled participants completed an online questionnaire (see S1 Text), capturing their sex, age, ethnicity, smoking status, previous COVID-19 testing, questions about their smell and taste symptoms, as well as other symptoms of COVID-19.

Participants were subsequently sent a point-of-care testing kit detecting the presence of immunoglobulin M (IgM) and immunoglobulin G (IgG) antibodies to SARS-CoV-2. A healthcare professional arranged a telemedicine video consultation with each participant. At the beginning of the consultation, they asked the participant to describe the changes that they had experienced in their sense of smell and/or taste. Participants were then supervised in performing the antibody test using a finger-prick sample of whole blood, and their results were discussed with them. Photographs of the test cassette were obtained and reviewed independently by a second healthcare professional to confirm the result. Participants with a prior COVID-19 PCR test result were offered antibody testing irrespective of their result. Testing was carried out between 24 April 2020 and 22 May 2020.

The antibody test used detects the presence of IgM and IgG antibodies to SARS-CoV-2 via lateral flow immunoassay (Wuhan UNscience Biotechnology Co., Ltd. COVID-19 Antibody IgM/IgG) [15]. As part of the test’s validation, 1,585 cases were tested: 421 (positive) clinically confirmed (including PCR) COVID-19 patients and 1,164 controls. These showed that the product has a relative sensitivity of 98.8% (95% CI 97.3%–99.6%) and a relative specificity of 98.0% (95% CI 97.15%–98.7%) [15].

The study received ethical approval from the National Health Service Queen’s Square Research Ethics Committee (IRAS Project ID 282668, ClinicalTrials.gov: NCT04377815) and was conducted in line with the declaration of Helsinki and Good Clinical Practice.

### Statistical analysis

A sample size calculation was undertaken in order to determine the study’s recruitment target, using the information on reported symptoms from the web-based COVID symptom study app developed by King’s College London and symptom reporting between the 24–29 March 2020 [16]. To calculate an estimate of 50% (95% CI 45%–55%) as the proportion who would test positive amongst those who reported a change in smell or taste, we needed 385 participants in the study [17]. Assuming a 15% attrition, we needed 453 participants in the study. The recruitment target was set to 500 participants to allow for larger attrition and increase accuracy. This was exceeded to improve the accuracy of the data and enable additional analyses to further describe the pattern of loss of smell and/or taste in the study populations with and without SARS-CoV-2 antibodies. Data were analyzed using GraphPad Prism version 8 (https://www.graphpad.com/scientific-software/prism/) and STATA version 15 (https://www.stata-uk.com/). Data analysis was planned on completion of SARS-CoV-2 antibody testing. There was no prospective study protocol or analysis plan, and no data-driven changes to analyses took place. Descriptive analyses included the calculation of means (plus standard deviation [SD]) for continuous variables and numbers (*n*, with percentages) for categorical variables. Chi-squared tests were performed on categorical data as part of the secondary analysis. The significance level was adjusted for multiple comparisons by applying a Bonferroni correction when comparing symptoms other than loss of smell or taste. Logistic regression analysis was performed to estimate the association between loss of smell and/or taste and the presence of SARS-CoV-2 antibodies.

## Results

### Study population

**Fig 1:**
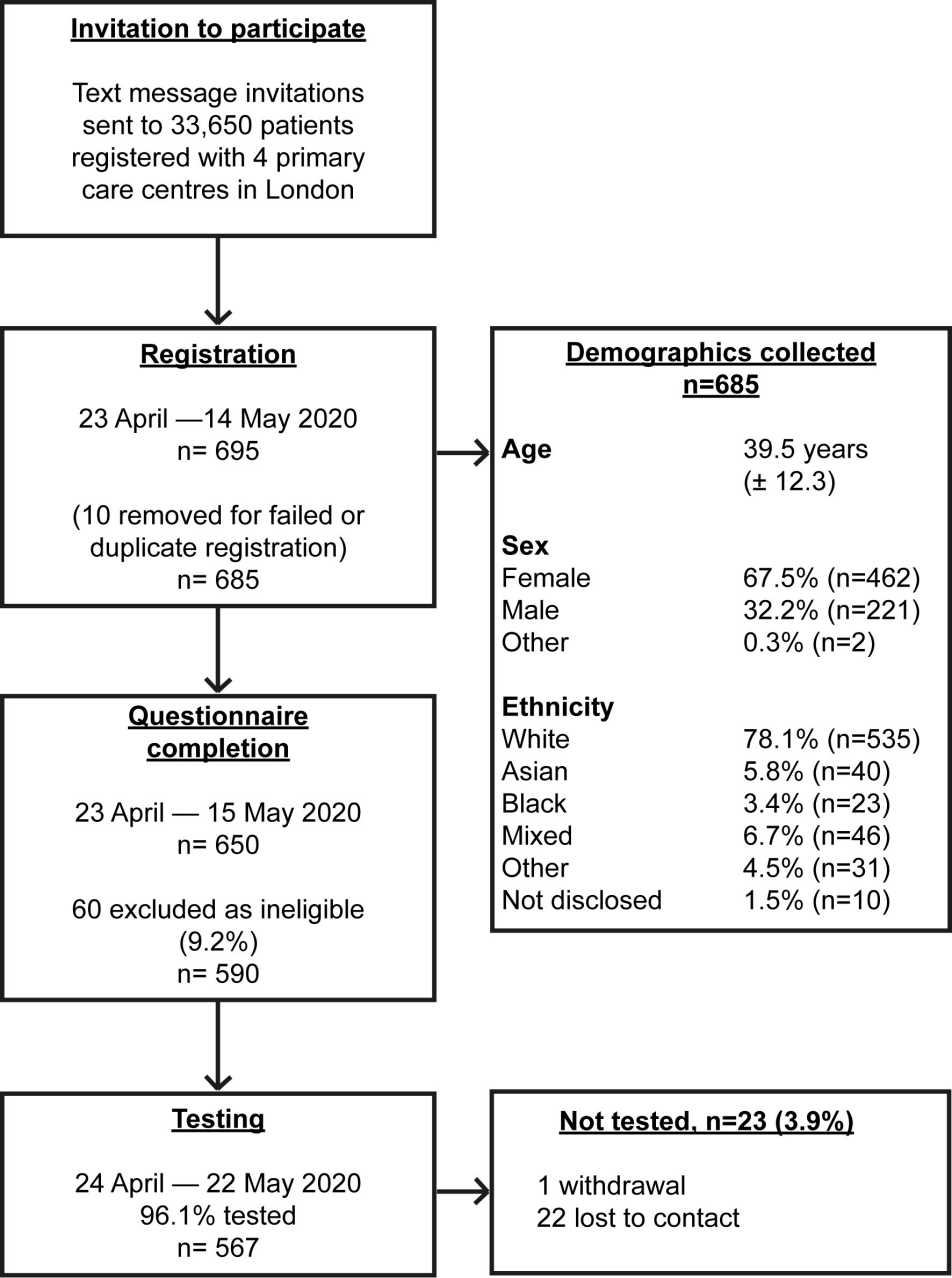
Participant flowchart illustrating participant flow through the recruitment process. Participant flowchart illustrating the participant flow through the recruitment process, from text invitations, through eligibility screening, questionnaire completion and testing [18]. Figures presented as % with total number (*n*). Age presented as mean age in years with standard deviation.

A total of 33,650 text messages were sent out to people registered with 4 participating primary care centers. A total of 650 participants completed the registration process; 60 participants were ineligible and excluded. The participant flowchart [18] is illustrated in Fig 1. Out of 590 eligible participants, 567 (96.1%) had a SARS-CoV-2 antibody test. The mean participant age was 39.4 years (±12.0); 69.1% (*n* = 392) were female, 30.5% (*n* = 173) male, and 0.4% (*n* = 2) of other sex. A total of (*n* = 311) 79.3% of female participants had a positive test result, compared with 73.4% (*n* = 127) of male participants (*p* = 0.120).

### Frequency of loss of sense of smell and taste in the study population

Among the 590 participants who completed questionnaires, 90.0% (*n* = 531) reported loss of their sense of smell. This was described as complete smell loss by 69.9% (*n* = 371) and as a partial smell loss by 30.1% (*n* = 160). No smell loss was reported in 10.0% (*n* = 59). A total of 89.8% (*n* = 530) of participants reported loss of their sense of taste. This was described as complete by 47.4% (*n* = 251) and as partial by 52.6% (*n* = 279). No taste loss was reported in 10.1% (*n* = 60) of participants. Combined loss of smell and taste was reported by 80.0% (*n* = 472).

### Seroprevalence of SARS-CoV-2 antibodies in people with acute loss of their sense of smell and/or taste

Of 590 eligible participants, 567 participants (96.1%) underwent SARS-CoV-2 testing. A total of 77.4% (*n* = 439) had a positive SARS-CoV-2 result (IgG [*n* = 303], IgG and IgM [*n* = 122], and IgM [*n* = 14]). One further participant was included with a positive PCR result (77.6% [*n* = 440] positive). Participants with and without SARS-CoV-2 antibodies were comparable in terms of age, sex, ethnicity, smoking status, and frequency of other reported symptoms (Table 2). Importantly, 52.1% (*n* = 229) of the participants with SARS-CoV-2 antibodies had no history of cough, and 39.8% (*n* = 175) had neither a fever nor a cough.

**Table 2:** Study group demographics by SARS-CoV-2 antibody test result.

Demographics	SARS-CoV-2 antibody positive (*n* = 440)	SARS-CoV-2 antibody negative (*n* = 127)	*p*-value
**Sex (%, with data)**			0.223
Female	70.7% (*n* = 311)	63.8% (*n* = 81)	
Male	28.9% (*n* = 127)	36.2% (*n* = 46)	
Other	0.5% (*n* = 2)	0.0% (*n* = 0)	
**Age (mean, SD, years)**	39.1 (±8.9)	40.7 (±9.8)	0.250
**Ethnicity (no, %, with data)**			0.754
White	79.5% (*n* = 350)	78.7% (*n* = 100)	
Asian	6.4% (*n* = 28)	5.5% (*n* = 7)	
Black	2.7% (*n* = 12)	1.6% (*n* = 2)	
Mixed	5.9% (*n* = 26)	7.1% (*n* = 9)	
Other	4.8% (*n* = 21)	4.7% (*n* = 6)	
Not disclosed	0.7% (*n* = 3)	2.4% (*n* = 3)	
**Smoking (%, with data)**			0.153
Current smokers	8.4% (*n* = 37)	12.6% (*n* = 16)	
Non-/Ex-smokers	91.6% (*n* = 403)	87.4% (*n* = 111)	
**Other symptoms** **(%, with data)**			
Cough	47.7% (*n* = 210)	48.0% (*n* = 61)	0.952
Fever	37.7% (*n* = 167)	25.2% (*n* = 32)	0.080
Shortness of breath	37.1% (*n* = 163)	33.9% (*n* = 43)	0.511
Headache	64.3% (*n* = 283)	63.0% (*n* = 80)	0.784
Sore throat	40.9% (*n* = 180)	52.0% (*n* = 66)	0.027
Hoarse voice	17.3% (*n* = 76)	21.3% (*n* = 27)	0.305
Chest pain and/or tightness	37.7% (*n* = 166)	31.5% (*n* = 40)	0.198
Abdominal pain	15.0% (*n* = 66)	24.4% (*n* = 31)	0.013
Diarrhea	25.6% (*n* = 117)	34.7% (*n* = 44)	0.076
Vomiting	3.6% (*n* = 16)	1.6% (*n* = 2)	0.243
Confusion, disorientation and/or drowsiness	32.3% (*n* = 142)	33.9% (*n* = 43)	0.737
Muscle and/or joint pain	60.2% (*n* = 265)	52.0% (*n* = 66)	0.096
Loss of appetite	57.3% (*n* = 252)	56.0% (*n* = 71)	0.784

Table comparing age, sex, ethnicity, smoking status as well as additional reported symptoms between participants with positive and negative SARS-CoV-2 antibodies. No significant differences were detected between the two groups, in any variable reported in this table. The significance level for other symptoms was adjusted for multiple comparisons to *p* = 0.004 through a Bonferroni correction.

SARS-CoV-2, severe acute respiratory syndrome coronavirus 2; SD, standard deviation).

### Frequency of loss of smell and taste in participants with and without SARS-CoV-2 antibodies

**Fig 2:**
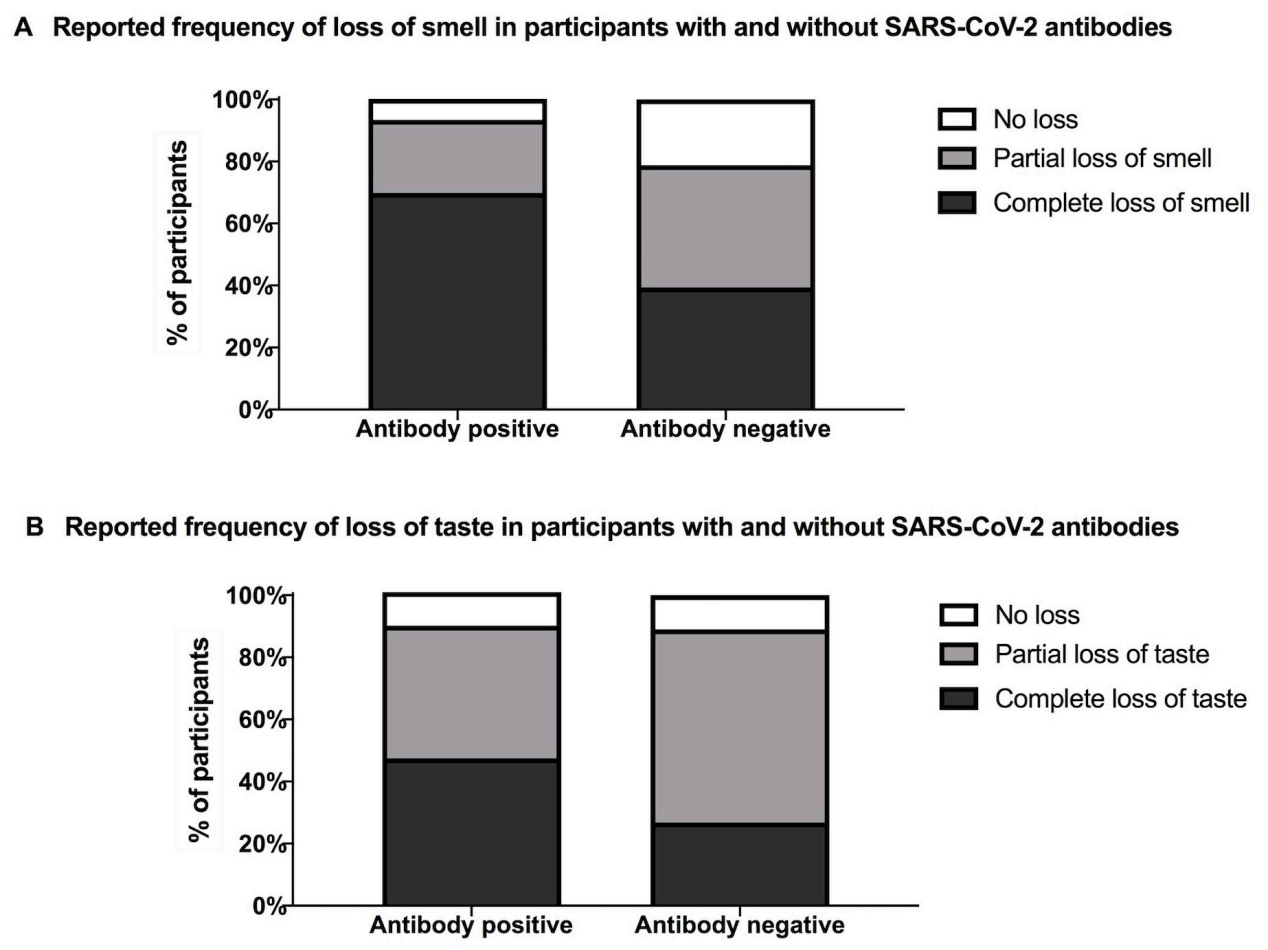
**Reported frequency of loss of smell (A) and loss of taste (B) in participants with and without SARS-CoV-2 antibodies. A**. Out of 440 participants with SARS-CoV-2 antibodies, 93.4% (*n* = 411) reported a loss of smell (complete loss of smell in 69.8%, *n* = 307 and partial loss of smell in 23.6%, *n* = 104) and 6.6% (*n* = 29) reporting no change. Out of 127 participants without SARS-CoV-2 antibodies, 78.7% (*n* = 100) reported a loss of smell (complete in 39.4%, *n* = 50 and partial in 39.4%, *n* = 50), and 21.2% (*n* = 27) reported no loss of smell. **B.** A total of 90.2% (*n* = 397) of participants with SARS-CoV-2 antibodies reported loss of taste (complete in 47.5%, *n* = 209 and partial by 42.7%, *n* = 188). No loss of taste was reported in 10.8% (*n* = 43) reported no loss of taste. In participants without SARS-CoV-2 antibodies, loss of taste was reported by 89.0% (*n* = 113) (complete in 26.8%, *n* = 34 and partial in 62.2%, *n* = 79). No loss of taste was reported in 11.0% (*n* = 14). A significantly greater percentage of participants with SARS-CoV-2 antibodies reported a loss of their sense of smell (93.4% versus 78.7%, *p* < 0.001) and complete loss of smell (69.8% versus 50%, *p* < 0.001) compared with participants without antibodies. SARS-CoV-2, severe acute respiratory syndrome coronavirus 2.

Among the participants who underwent SARS-CoV-2 antibody testing (*n* = 567), 89.9% (*n* = 510) reported loss of their sense of smell and 89.7% (*n* = 509) taste loss. The frequency of reported loss of smell and taste in participants with and without SARS-CoV-2 antibodies are presented in Fig 2 and Table 3. A total of 80.4% of participants with smell loss and 86.0% (*n* = 307) of those with complete smell loss had a positive test result. A total of 77.8% of participants with taste loss and 86.0% of those with complete taste loss (*n* = 209) had a positive test result. Extracts from questionnaire responses from participants with positive SARS-CoV-2 antibodies describing their loss of smell and taste can be seen in Table 4.

**Table 3:** Reported loss of sense of smell and/or taste in participants with positive and negative SARS-CoV-2 antibodies.

	SARS-CoV-2 antibody positive (*n* = 440)	SARS-CoV-2 antibody negative (*n* = 127)	*p*-value
**Sense of smell**			
Loss of the sense of smell (complete and partial)	93.4% (*n* = 411)	78.7% (*n* = 100)	*p* < 0.001
Complete loss of smell	69.8% (*n* = 307)	39.4% (*n* = 50)	*p* < 0.001
Partial loss of smell	23.6% (*n* = 104)	39.4% (*n* = 50)	*p* < 0.001
**Sense of taste**			
Loss of the sense of taste (complete and partial)	90.2% (*n* = 397)	89.0% (*n* = 113)	*p* = 0.738
Complete loss of taste	47.5% (*n* = 209)	26.8% (*n* = 34)	*p* <0.001
Partial loss of taste	42.7% (*n* = 188)	62.2% (*n* = 79)	*p* <0.001
**Combined loss of sense of smell and taste**			
Loss of sense of smell and taste (complete and partial)	83.6% (*n* = 368)	67.7% (*n* = 86)	*p* <0.001
Complete loss of both smell and taste	50.5% (*n* = 186)	20.5% (*n* = 26)	*p* < 0.001
Complete loss of smell, partial loss of taste	25.6% (*n* = 94)	15.7% (*n* = 20)	*p* = 0.060
Partial loss of smell, complete loss of taste	4.6% (*n* = 17)	3.9% (*n* = 5)	*p* = 0.642
Partial loss of both smell and taste	19.3% (*n* = 71)	27.6% (*n* = 35)	*p* < 0.001

SARS-CoV-2, severe acute respiratory syndrome coronavirus 2.

**Table 4:** Extracts from participants’ descriptions of their loss of smell and/or taste taken from questionnaire responses from participants with positive SARS-CoV-2 antibodies.

Examples of descriptions of participants’ loss of their sense of smell	Examples of descriptions of participants’ loss of their sense of taste
“Sense of smell vanished, couldn't smell anything from garlic to bleach to aromatherapy oils.”	“I could not taste even the spiciest of foods or sweetest. I tried different chillies too but nothing had a taste.”
“I lost my sense of smell but did not have a blocked nose which was very strange. I have to say I couldn't smell anything for roughly 14 days.”	“I could not taste anything. Including a large teaspoon of hot sauce.”
“I couldn't smell anything. The neighbours apartment caught on fire one night and if it wasn't for my flatmate (or the fire brigade later) I wouldn't have realised.”	”I could taste absolutely nothing. I tested various food and drink but absolutely nothing. I did not have a cold.”
“It was as if the nerves had fried. My nose was not blocked, I just suddenly was unable to smell anything.”	“I couldn't taste chillies or any food. Drinks were just liquid.”
“Zero smell. . .not even strong things like frying garlic and perfumes. It was unlike when I have had similar experiences with colds because my nasal passages were not blocked and I could breathe normally.”	“I could taste absolutely nothing. I tested various food and drink but absolutely nothing.”

SARS-CoV-2, severe acute respiratory syndrome coronavirus 2.

### Loss of smell predicts positive SARS-CoV-2 antibody status in a community-based population with an acute loss of their sense of smell and/or taste

Logistic regression was used to explore the relative importance of loss of smell and loss of taste, alone and in combination, as symptoms of COVID-19 infection, assessed by the presence of SARS-CoV-2 antibodies in our study population with acute loss of smell and taste. Isolated loss of smell and combined loss in the sense of smell and taste were compared with an isolated loss of taste (Table 5).

**Table 5:** Logistic regression exploring the seroprevalence of SARS-CoV-2 antibodies in people with loss in the sense of smell in isolation, loss in the sense of taste in isolation, and a loss both in the sense of smell and taste in combination.

	Odds ratio (95% CI) (unadjusted)	*p*-value		Odds ratio (95% CI) (adjusted)^1^	*p*-value
Loss in the sense of taste only (baseline)	1.00				
Loss in the sense of smell only	2.86 (95% CI 1.27–6.36)	<0.001	Loss in the sense of smell only	2.72 (95% CI 1.21–6.14)	0.016
Combined loss in the sense of smell and taste	3.98 (95% CI 2.24–7.08)	<0.001	Combined loss in the sense of smell and taste	4.11 (95% CI 2.29–7.37)	<0.001
Constant	1.07 (95% CI 0.64–1.81)	0.789	Constant	2.91 (0.75–11.35)	0.123

^1^For sex, age, ethnicity, and smoking status.

CI, confidence interval; SARS-CoV-2, severe acute respiratory syndrome coronavirus 2.

Participants with loss of smell alone were nearly 3 times more likely than participants with isolated taste loss to have SARS-CoV-2 antibodies (OR 2.86, 95% CI 1.37–6.36; *p* < 0.001), and participants with combined loss of smell and taste were 4 times more likely to have SARS-CoV-2 antibodies (OR 3.98, 95% CI 2.24–7.08; *p* < 0.001). These findings remained unchanged after adjusting for sex, age, ethnicity, and smoking status.

## Discussion

In this community-based cohort study, undertaken during the peak of the COVID-19 outbreak in London, the seroprevalence of SARS-CoV-2 antibodies in participants with new onset loss of sense of smell and/or taste, was 77.6%. A total of 39.8% (*n* = 175) of the study participants reported neither cough nor fever. In our study cohort, loss of smell was more prevalent in participants with SARS-CoV-2 antibodies compared with those without antibodies (93.4% versus 78.7%, *p* < 0.001), whereas taste loss was equally prevalent (90.2% versus 89.0%, *p* = 0.738). Furthermore, participants with acute smell loss were 3 times more to be seropositive for SARS-CoV-2 (OR 2.86; 95% CI 1.27–6.36; *p* < 0.001) compared with those with taste loss.

Loss of smell and taste were evaluated as separate symptoms, which enabled a direct comparison of their relationship to SARS-CoV-2 antibodies. Among participants, loss of sense of smell, but not taste, was significantly more prevalent in participants with SARS-CoV-2 antibodies, compared with those without. Moreover, within our study cohort with smell and taste loss, participants with loss of smell alone were 3 times more likely than participants with loss of taste alone to have SARS-CoV-2 antibodies. Participants who reported both a loss of smell and taste were 4 times more likely to have SARS-CoV-2 antibodies compared with participants with isolated taste loss. These findings suggest that a loss of smell is a highly specific symptom of COVID-19, in contrast to a loss of taste, despite their comparable frequency. Sense of taste and smell are interlinked, with retronasal olfaction being a major component of taste (flavor); thus, it is plausible that the loss of taste reported by participants who also report loss of smell reflects impaired retronasal olfaction and hence, represents loss of flavor perception, as a consequence of smell loss. Interestingly, 6.6*%* of participants with SARS-CoV-2 antibodies reported an isolated loss in their sense of taste, in the absence of smell loss, suggesting the presence of a rarer, alternative pathophysiological mechanism targeting gustatory function in isolation. Lingual mucosal epithelium ACE2 could provide a plausible explanation [19].

Globally, as populations are released from lockdown, early identification by the public of COVID-19 symptoms and rapid self-isolation and testing will be of vital importance to limit disease spread. Currently, as highlighted in Table 1, a large number of countries are not advising that loss of smell and/or taste are COVID-19 symptoms. This could have potentially devastating consequences. Importantly, 40% of our seropositive cohort reported neither fever nor cough. Similarly, a recent UK-based survey reported that cough and/or fever were only present in 51% of people with COVID-19 [3]. Counterintuitively, people with minor symptoms, such as isolated smell loss, who remain systemically well pose the highest public health threat.

SARS-CoV-2 specific antibody testing performed via telemedicine consultation enabled us to confirm participants’ reported symptomatology and verify their identity and test results, which adds to the quality of our data. Although viral nucleic acid detection using real-time PCR remains the gold standard for diagnosis, there are several limitations to this method, including a high processing time, labor intensity, and a high false negative rate [20–22]. Furthermore, there is a narrow window to perform testing before the virus becomes undetectable by PCR [23]. In contrast, seroconversion to IgM and IgG antibodies occurs as early as day 4 [24]. The Center for Disease Control and Prevention recommends antibody testing for diagnostic purposes using high specificity kits to minimize false positives [22]. Given the very high prevalence in our study cohort and the specificity of our chosen test, we could expect a high positive predictive value [22]. The sensitivity of our antibody test is 98.8%. These data were generated in people who were PCR positive, suggesting a very high seroconversion rate. However, evidence with regard to the rate of patients with confirmed COVID-19, who seroconvert to generate an antibody response remains limited. Current data suggest seroconversion rates of up to 100% in hospital patients, which may be related to disease severity but also time from symptom onset [20,25,26]. Although a recent study demonstrated a 99.4% seroconversion rate in hospital staff following a mild COVID-19 illness, in which 47.5% of the study population reported anosmia [27], no data are available yet reporting the rate of seroconversion in confirmed COVID-19 patients with anosmia.

### Limitations

The main limitation of our study is the lack of a general population control group without loss of smell and/or taste. The study only recruited participants who reported acute smell and/or taste loss. Although this enabled us to study this presentation and its relevance to COVID-19, this also presents a degree of selection bias; hence, our findings refer to a population subset with new acute loss of smell and/or taste. In addition, although we used a questionnaire to assess COVID-19 symptoms and subsequently validated participants’ responses during the telemedicine interview, we did not undertake any objective assessments of smell or taste. However, from a COVID-19 disease containment perspective, the general public are unlikely to have rapid access to formal objective smell/taste testing. Moreover, our data show a high seroprevalence of SARS-CoV-2 antibodies in people who recognized a loss of their sense of smell. Furthermore, as our study relied on antibody testing via telemedicine, we were unable to account for COVID-19 patients who may not have seroconverted to develop IgG or IgM antibodies, which may lead to our findings underestimating the prevalence of COVID-19 in this population. However, our findings suggest that a key public health message is that people who notice a loss in their ability to smell everyday house-hold odors such as garlic, onions, coffee, and perfumes should self-isolate and seek PCR testing.

The majority of our participants were female; this finding may reflect previous findings that females have a higher frequency of loss of smell and/or taste with COVID-19 than males [3,9,28]. We found no differences in sex, but this may reflect the lower number of males recruited.

## Conclusions

In a community-based population, the vast majority of participants with new onset loss of smell were seropositive for SARS-CoV-2 antibodies. Acute loss of sense of smell needs to be considered globally as a criterion for self-isolation, testing, and contact tracing in order to contain the spread of COVID-19.

## Supporting information

S1 STROBE checklist STROBE, Strengthening the Reporting of Observational Studies in Epidemiology. (DOCX)

S1 Text Questionnaires used to capture demographics (part 1) and symptoms in study participants (part 2). (DOCX)

S1 Data Study data set. (XLSX)

## Abbreviations

ACE-2: angiotensin-converting enzyme-2
COVID-19: coronavirus disease 2019
IgG: immunoglobulin G
IgM: immunoglobulin M
PCR: polymerase chain reaction
SARS-CoV-2: severe acute respiratory syndrome coronavirus 2
