# Supplementary material for: Seroprevalence of SARS-CoV-2 antibodies in people with an acute loss in their sense of smell and/or taste in a community-based population in London, UK: An observational cohort study
Source: PLoS Med. 2020 Oct 1;17(10):e1003358. doi: 10.1371/journal.pmed.1003358 (PMC7529306; doi:10.1371/journal.pmed.1003358)
Supplement: S1 Text — (DOCX) [file pmed.1003358.s002.docx]

**Supporting Information: Part 1: Participant demographics questions**

|  | Question | Answer field |
| --- | --- | --- |
| 1 | **Date of birth** | DD/MM/YYYY |
| 2 | **Sex** | Female  Male  Other |
| 3 | **Ethnicity** | White  Black  Asian  Mixed  Other  I do not wish to disclose |
| 4 | **Have you ever smoked?** | Current smoker  Ex-smoker  Never smoked |

**Supporting Information: Part 2: Participant symptom questionnaire**

|  | Question | Answer field | |
| --- | --- | --- | --- |
| 1 | **Have you noticed any changes in your sense of smell?** | Yes  No | |
| 1.2 | **How would you describe the change in your sense of smell?** | - I can/could no longer smell any odours that I used to be able to smell - I can/could no longer smell some odours I used to be able to smell  Describe (optional) | |
| 1.3 | **Have you noticed that odours smell different than they used to?** | No  Yes  If yes describe (optional) | |
| 2 | **Have you noticed any changes in your sense of taste?** | Yes  No | |
| 2.1 | **How would you describe the changes in your sense of taste?** | - I can/could no longer taste any foods or drinks - My ability to taste food or drinks is/was reduced Describe (optional) | |
| 2.2 | **Have you noticed that foods and drinks taste differently?** | Yes  No  If yes describe (optional) | |
| 2.3 | **Have you experienced any unusual tastes while not eating or drinking?** | Yes  No  If yes describe | |
| 3 | **Have you experienced any of these symptoms in the last 4 weeks?** | Cough | Yes/No |
|  |  | Fever (37.8◦C or more) |  |
|  |  | Shortness of breath |  |
|  |  | Headache |  |
|  |  | Sore throat |  |
|  |  | Hoarse voice |  |
|  |  | Chest pain/tightness |  |
|  |  | Abdominal pain |  |
|  |  | Diarrhoea |  |
|  |  | Vomiting |  |
|  |  | Confusion, disorientation or drowsiness |  |
|  |  | Muscle/joint aches |  |
| 4 | **Has/was your appetite for food decreased?** | Yes  No | |
| 5 | **Have you had a test for COVID-19?** | Yes  No  If Yes: positive negative Type of test: swab test blood test  Location of test | |
